# Impact of green credit policy on the operation of Vietnamese commercial banks: An empirical study using difference-in-differences model

**DOI:** 10.1371/journal.pone.0333759

**Published:** 2025-10-24

**Authors:** Minh Phuong Nguyen, Anh Phan

**Affiliations:** 1 Faculty of Banking, Banking Academy of Vietnam, Hanoi, Vietnam; 2 Banking Research Institute, Banking Academy of Vietnam, Hanoi, Vietnam; Massey University, NEW ZEALAND

## Abstract

Green credit is one of the important activities that commercial banks show their responsibility to the environment. In Vietnam, the Government and the State Bank of Vietnam have implemented numerous regulations pertaining to green credit in order to encourage and support environmentally-friendly financial activity. Against a backdrop of diverse and often conflicting perspectives on the economic implications of sustainable development and EGS principles, empirical analysis of green finance policies is critically important. The article examines the effects of the Green Credit Policy on commercial banks’ operations in Vietnam using panel data from financial statements and annual reports of commercial banks from 2012 to 2022. Specifically, the study focuses on changes in profits, cost management, non-performing loan and capital adequacy ratio. The analysis is conducted using difference in differences (DID) model, with the sample divided into groups that implemented the policy and groups that did not. The study’s findings indicate the following impacts: (1) there is no empirical evidence supporting the notion that the green credit policy increases the profitability of commercial banks, (2) there is no empirical evidence suggesting that the green credit policy reduces the cost-to-income ratio, (3) the implementation of the green credit policy does have a negative impact on non-performing loan ratio of banks. The research findings serve as the foundation for the authors to propose several suggestions for commercial banks and the State Bank of Vietnam.

## 1. Introduction

In the context of increasingly serious climate and environmental change, green credit has become an effective financial tool to minimize negative impacts on the environment, promote sustainable economic growth, and demonstrate environmental responsibility of financial institutions. In the world, green credit policies have brought many benefits to commercial banks and the economy in general. Recognizing the role of green credit, in March 2015, the State Bank of Vietnam (SBV) issued Directive No. 03/CT-NHNN to guide and promote the banking system to implement sustainable financial activities, supporting environmentally friendly projects and business activities. Previous studies primarily focused on the current state of green finance and green credit without thoroughly exploring green credit policies or examining the multi-dimensional impact of these policies on various aspects of commercial bank operations [1–8]. In this paper, we use the difference-in-differences (DID) model – a specialized method for evaluating policy effectiveness and gradually widely used in many fields to conduct research [9,10]. According to the Credit Department of Economic Sectors, SBV, as of December 31, 2023, the outstanding green credit balance in the banking sector increased by 24% compared to the end of 2022, accounting for about 4.5% of the total outstanding credit in the entire economy. For banks with low non-performing loan ratios such as BIDV, green credit outstanding also accounted for only about 4.5% of the bank’s total outstanding credit (BIDV, 2024). Looking back more than 8 years after implementing Directive No. 03/CT-NHNN issued by the SBV in March 2015 to promote green credit growth and manage environmental and social risks in credit activities, it can be seen that the growth rate of green credit in Vietnam has shown signs of improvement; however, the total outstanding green credit still represents a small proportion of total outstanding credit.

Using the updated data set from the annual reports and the audited consolidated financial statements of 14 listed commercial banks in Vietnam in the period from 2012 to 2022 with a total of 154 observations, we expect to find evidence of the impact of green credit policies on several key indicators in the operations of commercial banks. The results show that the green credit policy has reduced the non-performing loan ratio of commercial banks in Vietnam. Therefore, our research has contributed to the theoretical and practical basis system when clarifying the application of the DID model in policy impact analysis; providing reference material for future studies; contributing to raising awareness of the role of green credit policies; and offering several recommendations to the SBV and commercial banks. The remainder of this article is structured as follows: Section 2 provides theoretical basis and literature review; Section 3 hypothesis development and presents the research design; Section 4 presents the empirical regressions and results; Section 5 provides the conclusions and some recommendations.

## 2. Literature review

### 2.1. Theoretical background

**Green credit policy** The green credit policy includes a series of policies and institutions to promote activities to reduce environmental pollution and use energy efficiency through credit interventions. Lv et al. [11] argue that green credit policy relies on market mechanisms to solve environmental problems and is a new type of environmental governance program that rationally allocates credit through the Government’s policy constraints to promote green economic transformation and green development trends in the whole society. In summary, the green credit policy is a system of regulations and guidelines issued to encourage commercial banks to prioritize capital sources for environmentally friendly projects and business activities, contributing to environmental protection and sustainable development, including directives, decisions, e.t.c. regulations of the SBV and other state agencies related to the field of green credit. From there, the research paper will consider the impact of the green credit policy from the State Bank of Vietnam on the performance of commercial banks in the research stage.

**Operations of Commercial Banks in Key Aspects** Financial indicators play an important and irreplaceable role in evaluating the performance of commercial banks, thereby helping to analyze the financial situation, measure business efficiency, support rational management decision-making and ensure safety, e.t.c. stability of the banking system. On the basis of inheriting the results of previous studies, this study will apply some of the following financial indicators.

*Profit*s is a factor that directly affects the bank’s operation, is the index that best reflects the bank’s performance and is the ultimate goal of commercial banks [12]. In studies, profits are often measured by the natural logarithm of after-tax profits on the Earnings Statement [13]. When the bank’s operations achieve good results and achieve the profit target, it will ensure the important role of credit institutions in the intermediary financial system, thereby promoting economic development.

*The cost-to-income ratio (CIR)* plays an important role and has the opposite effect on the bank’s performance. Strengthening operational cost management will improve operational efficiency, thereby increasing profits for banks. Therefore, CIR is considered to have an adverse impact on the performance of commercial banks [14].

*The non-performing loan ratio (NPLR)* is often considered an important indicator to assess a bank’s financial health. NPLR represents the ratio of unrecoverable debts to total outstanding loans [15]. A low NPL ratio is often an indication that the bank is managing credit risk effectively and is able to respond to financial risks.

*The Capital Adequacy Ratio (CAR)* is very important for figuring out how well a bank can handle financial risks. It makes sure the firm can handle unexpected losses and follow international rules like Basel. Green credit programs can have a big effect on CAR by changing a bank’s loan portfolio and improving how it handles risk. This connection is very important for keeping the economy stable and following the rules. Green credit encourages banks to put money into projects that are good for the environment. This could lead to a more stable asset base, which could improve CAR [16]. Banks can lower their overall risk profile and CAR by focusing on loans that are good for the environment. This is because they are less likely to lend to high-risk sectors [17,18]

### 2.2. Overview of related studies

**The impact of Green Credit Policies on commercial banks’ profitability** Although many studies point to a positive relationship between green credit policies and bank profits, some studies conclude the opposite. For example, Wu and Shen [4] and Oikonomou et al. [19] argue that commercial banks deploy green credit to protect shareholder interests, attract investment, increase deposits, and share capital, and expand business and increase profits. Luo et al. [5] and Zhou et al. [20] also found that China’s green credit policy in 2012 improved the competitiveness and profitability of commercial banks. However, Scholtens & Dam [1] and Wright [3] argue that green credit has long project durations, large investments, and high policy risks, increasing costs and decreasing profits. Banks can lower lending rates to attract businesses and reduce short-term profits [21]. Song et al. [22] also found that green credit reduces bank performance in some areas. Therefore, the green credit policy may reduce the profitability of commercial banks.

**Impact of Green Credit Policy on the Cost-to-Income Ratio of commercial banks:** Current studies are also not consistent on the impact of green credit policies on CIR ratios. Some studies show that green banks are gradually shifting from focusing on shareholder interests to general interests, helping to improve social reputation and reduce transaction costs as well as brand costs. thereby reducing the cost-to-income ratio [23]. However, green credit can increase costs due to the high requirement for environmental technology assessment skills, and this cost can sometimes exceed the financial benefits [24,25]. Research by Xiaoyan and Yiyang [13] also shows that green credit policies do not reduce the cost-to-income ratio of banks. While green credit has a long-term positive impact in developing countries, its short-term effectiveness is affected by escalating costs. The cost-to-income ratio is still high due to large initial investment costs and the scale of green credit is not large enough.

**The impact of Green Credit Policy on Non-Performing Loan Ratio of commercial banks:** Many studies indicate that the green credit policy can the non-performing loan ratio (NPLR) of commercial banks, but there is also an opinion that it can increase this ratio. Cui et al. [26] found that increasing green loans helped reduce the NPLR of Chinese banks. Nguyet et al. [27] believe that green credit increases credit scale, income, and reduces NPLR, improving the operational efficiency of commercial banks. Wu [28] also emphasized the positive impact of green credit in reducing NPLR. However, Zhang et al. [29] point out that polluting enterprises can increase debt costs, and the transition to a green model is costly, leading to difficulty in repaying debts and increasing bad debt. In addition, to maximize profits, commercial banks can ignore environmental risks, increase loans to businesses with low credit ratings, leading to an increase in bad debts.

**Application of the DID model to assess the multidimensional impact of Green Credit policies on commercial bank operations:** The DID model plays an important role in assessing the multi-dimensional impact of green credit policies on commercial bank operations. Zhong et al. [30] used the DID model to analyze the impact of green credit on the performance of commercial banks in China. The results show that increasing revenue, reducing costs, and increasing the cost of penalties for commercial banks that do not implement policies can promote banks’ initiative in implementing green credit. The short-term effect of this policy is somewhat negative but gradually decreases over time. In the long term, green credit policies help commercial banks improve their reputation, increase the borrowing capacity of businesses and have a positive impact on profits and environmental protection. Based on data from 62 Chinese commercial banks in the period 2013–2020, Xiaoyan and Yiyang [13] analyzed the impact of green credit policies on commercial bank profits using the DID model. The results show that the green credit policy helps increase non-interest income, reduce the non-performing loan ratio and increase profits for commercial banks, especially those with low the non-performing loan ratio.

From the above studies, we have noticed certain gaps. Firstly, according to the understanding of the authors, their research paper is one of the first research papers to provide empirical evidence of the multidimensional impact of green credit policies on the operation of Vietnamese commercial banks. Previous studies in Vietnam related to this topic have been mainly qualitative, focusing on the current situation of green credit in Vietnam [6,8] without clarifying the multi-dimensional impact of green credit policies on the operation of Vietnamese commercial banks. Second, most previous studies have mainly looked at only one aspect of green credit policy [1–5] and have not analyzed the multi-dimensional impact of green credit policies on the operation of commercial banks. The authors’ research paper will delve into the analysis of the multi-dimensional impact of green credit policies on each specific aspect of the operation of Vietnamese commercial banks, including financial impacts, operational efficiency, risks, reflected through profits, etc the ratio of expenses to income and the ratio of bad debts. Third, we use the DID double difference model – a specialized measurement method for evaluating policy effectiveness and is gradually being widely used in many fields to conduct research [9,10]. The established DID economic model is more suitable for policy analysis than the traditional general regression method commonly used in other research papers.

## 3. Methodology

### 3.1. Research methodology

In this research paper, we use the Difference in Differences (DID) model, also known as the double difference model, which is a specialized measurement method used to assess policy effectiveness. According to Fredriksson and Oliveira [10], DID is one of the most frequently used methods in impact evaluation research. The DID model compares the change over time of a group that does not implement a policy with changes over time in the group that implements a policy, and the “difference in differences” rule is due to the impact of the policy. DID is often implemented as an interaction term between time and the dummy variables of the treatment group in regression models. Specifically, in the research paper, the DID model is utilized by selecting the variable TREAT*POST (described in detail in Table 1) as a dummy variable epresenting the green credit policy to analyze the impact of the green credit policy on the operations of commercial banks. However, the dependent variable can also be influenced by many other factors, in addition to the effects of policy and time. Therefore, we have included some control variables in the regression model (specifically in section 3.4 of the study). The value of the DID method is based on the premise that in the absence of a policy, the treatment and control groups would follow the same trend over time (the parallel assumption). This assumption is tested by graphing to assess the multidimensional impact of green credit policies on financial indicators reflecting the operations of banks, namely: profit, cost-to-income ratio, non-performing loan ratio and CAR of commercial banks. After checking the parallel assumptions, we proceeded to model regression to clarify the research hypotheses as well as the conclusions from the parallel assumptions.

**Table 1:** Description of the expected impact of independent variables.

Independent variable	Measure	Mark expectation	References
TREAT*POST	TREAT = 1 (Treatment group), TREAT = 0 (Control group) POST = 1 (from 2016 to 2024), in contrast, POST = 0 (before 2016)	+ (1) PROFIT	Luo et al. [5]; Zhou et al [20]
		– (2) CIR	Scholtens and Kang [23]; Wu and Shen [4]
		– (3) NPLR	Xiaoyan and Yiyang [13]; Cui et al. [26]; Nguyet et al. [27]; Wu [28]
		– (4) CAR	Lee et al. (2025)
SIZE	Ln (Total assets)	+	Vuong et al. [31]; Liu [32]
LDR	$\begin{array}{c} \frac{Total\;loans}{Total\;deposits} \end{array}$	+	Nguyen et al. [33]; Kolapo et al. [34]
NIM	$\begin{array}{c} \frac{Net\;interest\;income}{Average\;earning\;assets} \end{array}$	+	Pastory et al. [35]; Pasaman Silaban [36]; O’Connell [37]
GDP	GDP growth (annual %)	+	Dinh et al., 2025 [38]
INF	Inflation, consumer prices (annual %)	+	Dinh et al., 2025 [38]

### 3.2. Research data

According to the DID model, several data points need to be identified to measure the impact of green credit policies on the operations of commercial banks in Vietnam, including:

***Policy event 2016*** In Vietnam, in March 2015, the State Bank of Vietnam issued Directive No. 03/CT-NHNN “On Promoting Green Credit Growth and Managing Environmental and Social Risks in Credit Activities,” which stipulates that credit institutions actively implement the development of green credit programs and policies to gradually increase the proportion of green credit in their credit investment portfolios.In that same year, the Governor of the State Bank of Vietnam issued Decision No. 1552/QĐ-NHNN on August 6, 2015: “The action plan of the banking sector to implement the national strategy for green growth by 2020. With the goal of providing credit to green economic sectors, developing products and services to support businesses in achieving sustainable growth. Therefore, the regulations on implementing Green Credit by the State Bank of Vietnam are an urgent requirement for credit institutions. However, due to the inherent delays in policies, we have chosen the research milestone to be the year 2016. At the same time, according to the requirements of the DID method, the study period is a number of years before the policy intervention and all the years after its implementation.At the time of the study, commercial banks had not yet published their annual reports for 2025, therefore, we chose the research period from 2012 to 2024.

***Two groups of*** banks: the treatment group and the control group. The data in the study is secondary data collected from the financial statements and annual reports of 26 Vietnamese commercial banks, divided into two groups: the group that has implemented the policy and the group that has not implemented the policy (Table 2).

**Table 2:** Research bank list.

Banking Group	Bank Name
Treatment group	Saigon Thuong Tin Commercial Joint Stock Bank, Joint Stock Commercial Bank for Investment and Development of Vietnam, Ho Chi Minh City Development Joint Stock Commercial Bank, Joint Stock Commercial Bank for Foreign Trade of Vietnam, Saigon – Hanoi Commercial Joint Stock Bank, Asia Commercial Joint Stock Bank, Military Commercial Joint Stock Bank, Tien Phong Commercial Joint Stock Bank, Vietnam Joint Stock Commercial Bank for Industry and Trade, Vietnam Technological and Commercial Joint Stock Bank, Vietnam Prosperity Joint Stock Commercial Bank, Vietnam International Commercial Joint Stock Bank, Orient Commercial Joint Stock Bank, Vietnam Maritime Commercial Joint Stock Bank, Nam A Commercial Joint Stock Bank
Control group	Vietnam Export Import Commercial Joint Stock Bank, An Binh Commercial Joint Stock Bank, Bac A Commercial Joint Stock Bank, Viet Capital Commercial Joint Stock Bank, Kien Long Commercial Joint Stock Bank, Lien Viet Post Commercial Joint Stock Bank, National Citizen Commercial Joint Stock Bank, PG Bank, Saigon Bank for Industry and Trade, Vietnam Thuong Tin Commercial Joint Stock Bank

*Treatment group*: are the banks that fully, consistently, and transparently disclose the total amount of green credit data continuously over the years, have sustainability reports, and implement green credit in accordance with Directive No. 03 of the State Bank of Vietnam, specifically: researching and developing green credit products, implementing credit programs, prioritizing green credit allocation, having preferential lending policies for projects aimed at green growth, and having outstanding green credit loans during the research phase...

*Control group*:the banks have not yet, or have only modestly implemented green credit, with data on green credit being inconsistent over the years from 2012 to 2024. There is no outstanding green credit, and the implementation of green credit policies is negligible, as observed through announcements in annual reports, financial statements, and the websites of the banks.

### 3.3. Hypotheses

The theoretical analyses and empirical studies mentioned have found the impacts of green credit policies on the operations of commercial banks. However, the opinions are still not unified (Table 3). Therefore, we propose the following hypotheses:

**Table 3:** Studies on the multi-dimensional impact of green credit policies on the operation of commercial banks.

Affected factors	Co-directional impact	Reverse impact
Profitability	Choi and Pae [2]; Oikonomou et al. [19]; Wu and Shen [4]; Eisenbach et al. [39]; Luo et al. [5]; Zhou et al. [20]	Song et al. [22]
Cost-to-income ratio	Wright and Rwabizambuga [40]; Scholtens and Dam [1]; Wright [13]; Chen et al. [41]; Ehlers et al. [25]	Scholtens and Kang [23]; Wu and Shen [4]
Non-performing loan ratio	Ho [42]; Zhang et al. [29]; Zhou et al. [43]	Cui et al. [26]; Nguyet et al. [27]; Wu [28]
Capital Adequacy Ratio		Lee et al. [18]

*Hypothesis 1:* Green credit policies have an impact on increasing profits for commercial banks.

*Hypothesis 2*: The implementation of green credit policies affects the reduction of the cost-to-income ratio of commercial banks.

*Hypothesis 3:* The implementation of green credit policies impacts the non-performing loan ratio of commercial banks*.*

*Hypothesis 4:* The implementation of green credit policies improves banks’ capital adequacy ratio.

### 3.4. Research design

Refer to the research of Choi and Pae [2]; Oikonomou et al. [19]; Wu and Shen [4]; Eisenbach et al. [39]; Chen et al. [41]; Cui et al. [26]; Luo et al. [5]; Zhou et al. [20], Ehlers et al. [25]; Dang et al. [44]; Wu [28] and the authors selected the research model as well as put the appropriate variables into the general research model based on the DID theory as follows:


$$Y_{ik}=\beta_{\text{1}}TREAT*POST+\beta_{\text{2}}{GDP}_{t}+{\beta_{\text{3}}INF}_{t}\;+\sum \beta_{k}X_{kit}+\lambda_{i}+\varepsilon_{it}$$

With $Y_{ik}$ being profit, cost-to-income ratio, and non-performing loan ratio. Specifically:

Based on *Hypothesis 1, 2, 3 and 4,* we construct the following four regression equations for empirical analysis, respectively:


$${PROFIT}_{ik}=\beta_{\text{1}}TREAT*POST+\beta_{\text{2}}{GDP}_{t}+{\beta_{\text{3}}INF}_{t}+\;\sum \beta_{k}X_{kit}+\lambda_{i}+\varepsilon_{it}$$ (1)

$${CIR}_{ik}=\beta_{\text{1}}TREAT*POST+\beta_{\text{2}}{GDP}_{t}+{\beta_{\text{3}}INF}_{t}+\sum \beta_{k}X_{kit}+\lambda_{i}+\varepsilon_{it}$$ (2)

$${NPLR}_{ik}=\beta_{\text{1}}TREAT*POST+\beta_{\text{2}}{GDP}_{t}+{\beta_{\text{3}}INF}_{t}+\sum \beta_{k}X_{kit}+\lambda_{i}+\varepsilon_{it}\;$$ (3)

$${CAR}_{ik}=\beta_{\text{1}}TREAT*POST+\beta_{\text{2}}{GDP}_{t}+{\beta_{\text{3}}INF}_{t}+\sum \beta_{k}X_{kit}+\lambda_{i}+\varepsilon_{it}$$ (4)

In which:

λi represents individual fixed effects, where the index i represents the bank;

X represents the control variable; ε_it_ is the error of the regression model;

ε_**it**_ is the error of the regression model;

TREAT indicates whether the bank implements a green credit policy;

POST indicates whether the observation is before or after the policy year;

(1) PROFIT is the dependent variable indicating the profit of the commercial bank;
(2) CIR is the dependent variable that indicates the cost-to-income ratio of commercial banks;
(3) NPLR is the dependent variable indicating the non-performing loan ratio of commercial banks.
(4) *CAR* is the dependent variable indicating the Banks’ capital adequacy ratio.

## 4. Results and discussion

### 4.1. Descriptive statistics

The dataset comprises 26 commercial banks in Vietnam over the period from 2012 to 2024, yielding 284 observations across 11 years. Table 4 presents the descriptive statistics for the variables in the model, including the mean, standard deviation (SD), minimum (Min), and maximum (Max) values. The descriptive statistics provide a comprehensive overview of the variables used in the study. To minimize the influence of outliers on the regression results, we used the Winsorizing method. Specifically, dependent variables and control variables with values greater than the 99th percentile and less than the 1st percentile were replaced with the values at that percentile. This method helps eliminate extreme values while retaining all observations, ensuring the robustness of the model. The dependent variable PROFIT, measured as the natural logarithm of after-tax profits, has a mean of 5.38 × 10^12^ with a standard deviation of 7.35x10^12^ indicating moderate variability across the sample. The range (1.23 x 10^9^ to 3.19 x 10^13^) suggests that profitability levels among the banks vary significantly, potentially reflecting differences in bank size, operational efficiency, or market conditions over the study period.

**Table 4:** Descriptive statistics.

Variable	Obs	Mean	Std. Dev.	Min	Max
PROFIT w	284	5.380e + 12	7.353e + 12	1.238e + 09	3.198e + 13
CIR	284	.495	.16	.227	1.722
NPLR	284	.022	.022	.005	.298
CAR	284	.125	.034	.083	.334
TREATPOST	284	.426	.495	0	1
GDP	284	6.035	1.804	2.554	8.538
INF	284	3.537	1.789	.631	9.095
TA w	284	3.588e + 14	4.453e + 14	1.582e + 13	2.121e + 15
LDR	284	.756	.113	.463	1.122
NIM	284	.034	.013	.007	.094

The cost-to-income ratio (CIR) exhibits a mean of 0.495, with a relatively high standard deviation of 0.16, indicating substantial variation in cost efficiency among the banks. The wide range (0.22 to 1.72) highlights that some banks face significantly higher operational costs relative to their income, possibly due to inefficiencies or initial investments in green credit programs, particularly for the treatment group post-2016. This variability underscores the need to control for bank-specific factors in the regression analysis. The non-performing loan ratio (NPLR) has a mean of 0.022 with a standard deviation of 0.022, reflecting moderate dispersion in credit risk across the sample. The range (0.005 to 0.096) indicates that while some banks maintain very low NPLR, others face significant credit risk, potentially influenced by their exposure to green credit policies or broader economic conditions. Regarding the Capital Adequacy Ratio (CAR), a mean of 0.125 with a standard deviation of 0.034, the minimum CAR is 0.083 and the maximum is 0.334 suggest that all banks in the sample meet the minimum capital requirements, though there is some variation in their capital buffers.

The dummy variable TREAT*POST, representing the interaction between the treatment group and the post-policy period, has a mean of 0.426, indicating that approximately 42.6% of the observations pertain to the treatment group after 2016. This distribution suggests a relatively balanced sample between pre- and post-policy periods, which is essential for the DID methodology.

Among the control variables, total assets have a mean of 3.588 x 10^14^, with a narrow standard deviation of 4.45x10^14^, suggesting that the sample includes a diverse mix of small, medium, and large banks. The loan-to-deposit ratio (LDR) averages 0.756, with a standard deviation of 0.113, indicating moderate variation in liquidity management practices. The net interest margin (NIM) shows a mean of 0.034, with a standard deviation of 0.013.

These descriptive statistics highlight the heterogeneity in financial performance and operational characteristics among Vietnamese commercial banks, setting the stage for a robust analysis of the green credit policy’s impact using the DID model. The observed variability in PROFIT, CIR, CAR and NPLR, in particular, suggests that the policy may have differential effects across banks, which will be further explored in the regression analysis.

### 4.2. Relevant inspections

The results in Table 5 show the correlation coefficients between the independent variables and control variables in the three regression models, while Table 6 presents the multicollinearity test using the Variance Inflation Factor (VIF).

**Table 5:** Correlation coefficients between variables of the Models (1), (2), (3) and (4).

Variables	(1)	(2)	(3)	(4)	(5)	(6)	(7)	(8)	(9)	(10)
(1) ln_profit	1.000									
(2) CIR	−0.775	1.000								
	(0.000)									
(3) NPLR	−0.396	0.479	1.000							
	(0.000)	(0.000)								
(4) CAR	−0.301	0.084	0.071	1.000						
	(0.000)	(0.160)	(0.234)							
(5) TREATPOST	0.660	−0.511	−0.184	−0.255	1.000					
	(0.000)	(0.000)	(0.002)	(0.000)						
(6) ln_ta	0.844	−0.579	−0.174	−0.508	0.662	1.000				
	(0.000)	(0.000)	(0.003)	(0.000)	(0.000)					
(7) LDR	0.475	−0.430	−0.125	−0.015	0.334	0.405	1.000			
	(0.000)	(0.000)	(0.035)	(0.806)	(0.000)	(0.000)				
(8) NIM	0.390	−0.448	−0.068	0.254	0.256	0.153	0.373	1.000		
	(0.000)	(0.000)	(0.256)	(0.000)	(0.000)	(0.010)	(0.000)			
(9) GDP	−0.016	0.058	0.010	0.078	−0.029	−0.017	0.057	0.039	1.000	
	(0.794)	(0.328)	(0.864)	(0.192)	(0.621)	(0.777)	(0.335)	(0.511)		
(10) INF	−0.087	0.113	0.147	0.263	−0.206	−0.175	−0.119	0.010	0.017	1.000
	(0.145)	(0.056)	(0.013)	(0.000)	(0.000)	(0.003)	(0.046)	(0.860)	(0.776)	

**Table 6:** Multicollinearity test.

Variable name	VIF	1/VIF
Ln Total Assets	1.935	.517
TREATPOST	1.896	.527
LDR	1.37	.73
NIM	1.214	.824
INF	1.057	.946
GDP	1.007	.993
Mean VIF	1.413	.

The correlation analysis in Table 5 indicates that the relationships between the independent variables (TREATPOST, LDR, NIM, SIZE, GDP, INF) and the dependent variables (PROFIT, CIR, NPLR and CAR) are generally weak to moderate. This suggests a low likelihood of autocorrelation among the explanatory variables, ensuring the reliability of the regression models. Specifically, TREATPOST shows a moderate positive correlation with PROFIT (0.66, p < 0.0001) and Total assets (0.662, p < 0.0001), indicating that banks implementing the green credit policy post-2016 tend to have higher profitability and larger asset sizes, which aligns with expectations given the scale advantages of larger banks. However, TREATPOST has a negative correlation with NPLR (−0.184, p < 0.0001), suggesting that the policy may contribute to reducing non-performing loans, consistent with Hypothesis 3. The correlation between TREATPOST and CIR is negligible (−0.511, < 0.0001), which will be further tested in the regression.

The control variables also exhibit expected relationships. Total assets and PROFIT show a strong positive correlation (0.844, p < 0.0000), indicating that larger banks tend to have higher profitability, likely due to economies of scale and greater market presence. LDR and PROFIT are moderately correlated (0.457, p < 0.0000), suggesting that banks with higher loan-to-deposit ratios may generate more interest income, boosting profitability.

The multicollinearity test in Table 6 further confirms the robustness of the model. All VIF values are well below the threshold of 10, with the highest being 1.935 for total assets and the mean VIF at 1.413. This indicates no significant multicollinearity among the independent variables, ensuring that the regression coefficients are not distorted by inter-variable relationships. These diagnostic tests validate the model specification for the DID analysis. The absence of autocorrelation and multicollinearity ensures that the regression results will provide unbiased and reliable estimates of the green credit policy’s impact, allowing for meaningful interpretation of the policy’s effects on bank performance.

### 4.3. Parallel trend testing

The DID methodology was employed to assess the impact of the green credit policy, implemented in 2016, on the financial performance of commercial banks, with the parallel trends assumption being a critical prerequisite. This assumption requires that the treatment (implementing) and control (non-implementing) groups exhibit similar trends in key financial indicators prior to the policy intervention. This section evaluates the parallel trends for profitability, CIR, NPLR and CAR based on data from 2012 to 2024, with pre-policy trends analyzed from 2012 to 2015 and post-policy effects observed from 2017 to 2024.

**Fig 1:**
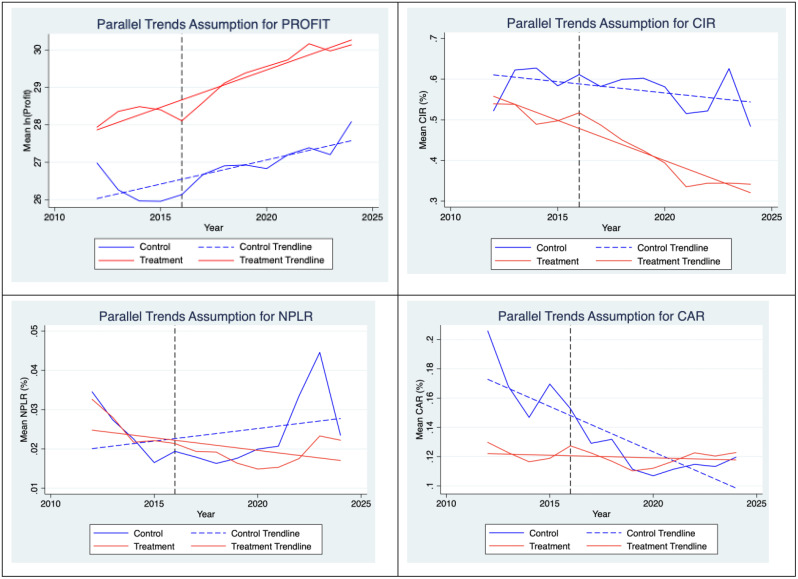
Parallel trends assumption test.

Fig 1 reveals that for profitability, both groups displayed a consistent upward trend prior to 2016 and post-2016, profitability continued to rise for both groups with minimal deviation between them. This stability suggests that the green credit policy had a limited impact on bank profitability, likely due to a balance between initial costs and revenues from green lending. The CIR before 2016 exhibiting a parallel trend that supports the DID framework. After 2016 the trend line of the experimental group tends to decrease faster than that of the control group. This provides visual evidence that the policy may have had a positive impact in helping banks reduce their cost-to-income ratio. Regarding the NPLR, for pre-2016 period, the trendlines for both groups show signs of non-parallelism. The trendline of the treatment group (red) has a slight upward trend, while the trendline of the control group (blue) is relatively flat. This shows that the trends of the two groups were different even before the policy took effect. Although the parallel trends assumption is not perfectly satisfied, the significant change in the trends of the two groups after the policy still provides an intuitive basis for its impact. For Capital Adequacy Ratio, the trend line of the control group (red) has a steeper slope and rises much faster than the trend line of the control group (blue) for pre-2016 period. This shows that these two groups of banks have had different trends in capital adequacy ratio (CAR) even before the policy took effect. In post-2016 period, the trend line of the treatment group continues to rise at a higher rate, while the control group also rises but at a slower rate. Since the parallel trend assumption is not met, CAR variable in the Difference-in-Differences regression model may lead to unreliable results, so the authors will remove this dependent variable, rejecting Hypothesis 4.

These preliminary insights will be further tested through regression analysis in Section 4.4 to determine the statistical significance of the policy’s effects and to validate the hypotheses outlined in Section 3.3.

### 4.4. Regression results

At first, Ordinary Least Squares (OLS), Fixed Effects (FEM), and Random Effects (REM) models are used to make our estimates. A number of diagnostic tests were conducted to make sure the models’strength. These tests showed that there was both heteroskedasticity and autocorrelation, which are common problems with panel data and can cause estimates to be less accurate. So, to deal with these data problems, the Feasible Generalized Least Squares (FGLS) model was chosen as the best way to estimate all of the regression equations. FGLS is the best and most reliable method for our investigation because it was made to give accurate and consistent estimates even when there is heteroskedasticity and autocorrelation.

The regression results for Equation (1) examine the impact of the green credit policy on profitability (PROFIT) (Table 7). The coefficient of TREAT*POST is 0.0688 with a t-statistic of 0.072, indicating that it is not statistically significant (p > 0.1). This suggests that there is insufficient evidence to conclude that the green credit policy increases the profitability of banks in the treatment group, thereby rejecting Hypothesis 1. This finding aligns with the parallel trend test in Section 4.3, which showed minimal divergence in profitability trends between the treatment and control groups post-2016. The lack of impact may be attributed to the small proportion of green credit in total outstanding loans (approximately 4% as of 2022, according to SBV [45]), which limits its influence on overall profitability. This result contrasts with studies such as Luo et al. [5] and Zhou et al. [20], who found that China’s green credit policy in 2012 improved the competitiveness and profitability of commercial banks by enhancing non-interest income and operational efficiency. However, our finding is consistent with Song et al. [22], who argued that green credit policies can reduce bank profitability in certain contexts due to high initial costs and long payback periods of green projects. The discrepancy may stem from the differing scales of green credit adoption, as China’s green credit market is more developed compared to Vietnam’s nascent stage. Among the control variables, total assets (1.192, p < 0.01) indicating that larger tend to be more profitable, likely due to economies of scale and greater interest income.

**Table 7:** Impact of green credit policy on profits, cost-to-income ratio, and non-performing loan ratio of commercial banks.

	(1)	(2)	(3)
	PROFIT	CIR	NPLR
TREATPOST	0.0688	0.00907	−0.00290^**^
	(0.0726)	(0.0106)	(0.00132)
ln_TA	1.192^***^	−0.0698^***^	−0.00207^***^
	(0.0441)	(0.00652)	(0.000755)
LDR	0.188	−0.0372	0.00344
	(0.326)	(0.0482)	(0.00546)
NIM	34.49^***^	−5.136^***^	−0.0970
	(3.312)	(0.525)	(0.0630)
GDP	−0.00219	0.00296^**^	−0.0000853
	(0.00936)	(0.00129)	(0.000163)
INF	0.0226^*^	−0.000167	0.00120^***^
	(0.0128)	(0.00191)	(0.000228)
_cons	−12.23^***^	2.944^***^	0.0855^***^
	(1.437)	(0.214)	(0.0246)
*N*	284	284	284

Standard errors in parentheses

*p < 0.1, ** *p* < 0.05, *** *p* < 0.01

For Equation (2), which assesses the impact on the CIR, the TREAT*POST coefficient is 0.00907 with a t-statistic of 0.0106, indicating no statistical significance (p > 0.1). This result implies that there is no evidence to suggest that the green credit policy affects the CIR of Vietnamese commercial banks, leading to the rejection of Hypothesis 2. The lack of a sustained effect may reflect the initial costs of policy adoption (e.g., training, technology upgrades) being offset by subsequent operational adjustments as banks adapt to green credit requirements. This result differs from Scholtens and Kang [23] and Wu and Shen [4], who suggested that green credit policies can reduce CIR by enhancing social reputation and lowering transaction costs. However, our finding aligns with Wright and Rwabizambuga [40], Scholtens and Dam [1], and Wright [3], who argued that green credit policies often increase operational costs due to the need for specialized skills in environmental risk assessment and compliance with regulatory requirements. The lack of a significant reduction in CIR in Vietnam may be attributed to the limited scale of green credit (4% of total loans) and the high initial investment costs, as noted by Ehlers et al. [25]. Among the control variables, total assets (−0.069, p < 0.01) has significant negative effect on CIR, suggesting that larger banks are more cost-efficient, possibly due to better resource management and revenue generation, a finding supported by Mathuva [14].

Equation (3) evaluates the impact on the NPLR. The TREAT*POST coefficient is −0.0029 and is statistically significant at the 5% level (t-statistic = −0.00132), indicating that the green credit policy significantly reduces the NPLR of banks in the treatment group. This result supports Hypothesis 3 and aligns with the parallel trend test in Section 4.3, which showed a sustained reduction in NPLR for the treatment group post-2016. The negative coefficient suggests that green credit policies encourage financing for environmentally friendly projects, which are often subject to rigorous risk assessments, leading to improved loan quality and lower credit risk. This finding is consistent with prior studies by Cui et al. [26], Nguyet et al. [27], and Wu [28], who highlighted the positive role of green credit in reducing NPLR by improving borrower quality and enforcing stricter credit standards. For instance, Cui et al. [26] found that increasing green loans helped reduce the NPLR of Chinese banks by focusing on sustainable projects with lower default risks. Similarly, Nguyet et al. [27] argued that green credit increases income and reduces NPLR in Vietnamese banks by enhancing operational efficiency. However, our result contrasts with Zhang et al. [29], who suggested that green credit policies might increase NPLR by raising debt costs for firms transitioning to green models, potentially leading to repayment difficulties. The positive outcome in Vietnam may be due to the careful selection of green projects and the SBV’s regulatory oversight, which mitigates such risks. Among the control variables, Total Assets (−0.00207, p < 0.01) also has significant negative effects on NPLR, indicating that larger banks manage credit risk more effectively, possibly due to better risk management practices and diversified loan portfolios, a finding supported by Nguyen et al. [33] and Kolapo et al. [34]. NIM’s coefficient (−0.097) is not significant (p > 0.1), suggesting that interest margins do not play a substantial role in reducing NPLR.

The regression results reveal a nuanced impact of the green credit policy on Vietnamese commercial banks, with findings that both align with and diverge from prior literature. The lack of a significant effect on profitability and CIR suggests that the policy’s financial benefits are not yet pronounced, possibly due to the nascent stage of green credit adoption in Vietnam, where green loans constitute a small fraction of total credit. This contrasts with more developed markets like China, where green credit has been shown to enhance profitability [5,20]. However, the significant reduction in NPLR highlights a key benefit of the policy in enhancing credit quality, consistent with findings in both Chinese [26] and Vietnamese [27] contexts. The control variables consistently show that bank size (SIZE) is a critical determinant of performance, positively affecting profitability while reducing CIR and NPLR, underscoring the advantages of scale in banking operations. The significant effects of LDR on all three indicators suggest that liquidity management plays a pivotal role in financial performance, with higher loan-to-deposit ratios linked to better profitability and lower CIR and NPLR, though this may also reflect increased risk exposure that requires careful monitoring. These findings provide a foundation for the policy recommendations in Section 5, particularly in leveraging the policy’s success in reducing NPLR to promote sustainable banking practices while addressing the challenges in profitability and cost efficiency.

## 5. Conclusions and recommendations

This study utilized the DID methodology to examine the impact of the green credit policy, implemented in 2016, on the operations of Vietnamese commercial banks. The parallel trend testing in Section 4.3 confirmed the validity of the DID approach, showing that pre-policy trends in profitability, CIR, and NPLR were similar between the treatment and control groups. The regression results in Section 4.4 further revealed that there is no empirical evidence to support the notion that the green credit policy increases profitability (rejecting Hypothesis 1) or reduces the CIR (rejecting Hypothesis 2) of commercial banks. However, the policy significantly reduces the NPLR (supporting Hypothesis 3), indicating a positive impact on credit quality. The limited impact on profitability and CIR may be attributed to the small proportion of green credit in total outstanding loans (approximately 4% as of 2022, according to SBV [45]), which restricts its influence on overall financial performance. Despite this, the reduction in NPLR underscores the policy’s potential to enhance financial stability and support sustainable development in the banking sector.

The findings have several practical implications for policymakers and commercial banks in Vietnam. First, the SBV should continue to develop and refine the legal framework for green credit, incorporating specific regulations and guidelines on environmental and social risk management to amplify the policy’s impact on the economy through banking credit channels. The SBV could introduce incentives such as reducing the mandatory reserve ratio for capital mobilized for green projects, offering refinancing incentives, or providing rediscounts for green loan portfolios. Additionally, coordination with relevant ministries and sectors is essential to address challenges in implementing green credit activities effectively.

Second, commercial banks should develop internal policies aligned with green objectives, focusing on the research, deployment, and development of green banking products and services to minimize environmental impacts. Preferential lending policies, such as reduced interest rates and extended loan terms, could encourage environmentally friendly projects. Banks should also invest in modern banking services, including software for managing and analyzing debt quality, and proactively seek green capital from international financial institutions, NGOs, or green finance trusts to support sustainable projects.

Third, capacity building is crucial. Commercial banks should organize training programs to enhance the awareness and expertise of staff in green credit, particularly in assessment, appraisal, and supervision of green loan packages. Furthermore, banks should collaborate with the SBV and other stakeholders to raise public awareness of green growth, fostering greater demand for green credit and supporting its development in Vietnam.

The study’s findings contribute to the literature by providing empirical evidence of the multidimensional impact of green credit policies on Vietnamese commercial banks, an area previously underexplored in the Vietnamese context. The significant reduction in NPLR highlights the policy’s role in promoting sustainable banking practices, aligning with Vietnam’s broader goals of green and sustainable economic growth. However, the research has limitations: (1) the sample size is limited due to challenges in accessing financial statements and annual reports of some banks, and (2) there is an imbalance between the treatment and control groups, as most banks have adopted green credit policies to some extent due to SBV requirements. Future research could expand the sample size, incorporate additional financial indicators, and explore the long-term effects of green credit as its adoption scales up in Vietnam.
